# Microenvironment in neuroblastoma: isolation and characterization of tumor-derived mesenchymal stromal cells

**DOI:** 10.1186/s12885-018-5082-2

**Published:** 2018-11-27

**Authors:** Gloria Pelizzo, Veronica Veschi, Melissa Mantelli, Stefania Croce, Vincenzo Di Benedetto, Paolo D’Angelo, Alice Maltese, Laura Catenacci, Tiziana Apuzzo, Emanuela Scavo, Antonia Moretta, Matilde Todaro, Giorgio Stassi, Maria Antonietta Avanzini, Valeria Calcaterra

**Affiliations:** 1 Pediatric Surgery Department, Children’s Hospital G. Di Cristina, ARNAS Civico-Di Cristina-Benfratelli, Via dei Benedettini n.1, 90134 Palermo, Italy; 2 Cellular and Molecular Pathophysiology Laboratory, Department of Surgical, Oncological and Stomatological Sciences, University of Palermo, Palermo, Italy; 3 Immunology and Transplantation Laboratory, Cell Factory, Pediatric Hematology Oncology Unit, Department of Maternal and Children’s Health, Fondazione IRCCS Policlinico S. Matteo, Pavia, Italy; 4 Pediatric Surgery Unit and NICU Policlinico-Vittorio Emanuele Hospital, Catania, Italy; 5 Pediatric Hematology Oncology Unit, Children’s Hospital G. Di Cristina, ARNAS Civico-Di Cristina-Benfratelli, Palermo, Italy; 6 Department of DIBIMIS, University of Palermo, 90127 Palermo, Italy; 7 Pediatrics and Adolescentology Unit, Department of Internal Medicine, University of Pavia, Fondazione IRCCS Policlinico San Matteo, Pavia, Italy

**Keywords:** Neuroblastoma, Mesenchymal stromal cells, Microenvironment, Stemness, EMT, Children

## Abstract

**Background:**

It has been proposed that mesenchymal stromal cells (MSCs) promote tumor progression by interacting with tumor cells and other stroma cells in the complex network of the tumor microenvironment. We characterized MSCs isolated and expanded from tumor tissues of pediatric patients diagnosed with neuroblastomas (NB-MSCs) to define interactions with the tumor microenvironment.

**Methods:**

Specimens were obtained from 7 pediatric patients diagnosed with neuroblastoma (NB). Morphology, immunophenotype, differentiation capacity, proliferative growth, expression of stemness and neural differentiation markers were evaluated. Moreover, the ability of cells to modulate the immune response, i.e. inhibition of phytohemagglutinin (PHA) activated peripheral blood mononuclear cells (PBMCs) and natural killer (NK) cytotoxic function, was examined. Gene expression profiles, known to be related to tumor cell stemness, Wnt pathway activation, epithelial-mesenchymal transition (EMT) and tumor metastasis were also evaluated. Healthy donor bone marrow-derived MSCs (BM-MSC) were employed as controls.

**Results:**

NB-MSCs presented the typical MSC morphology and phenotype. They showed a proliferative capacity superimposable to BM-MSCs. Stemness marker expression (Sox2, Nanog, Oct3/4) was comparable to BM-MSCs. NB-MSC in vitro osteogenic and chondrogenic differentiation was similar to BM-MSCs, but NB-MSCs lacked adipogenic differentiation capacity. NB-MSCs reached senescence phases at a median passage of P7 (range, P5-P13). NB-MSCs exhibited greater immunosuppressive capacity on activated T lymphocytes at a 1:2 (MSC: PBMC) ratio compared with BM-MSCs (*p* = 0.018). NK cytotoxic activity was not influenced by co-culture, either with BM-MSCs or NB-MSCs. Flow-cytometry cell cycle analysis showed that NB-MSCs had an increased number of cells in the G0-G1 phase compared to BM-MSCs. Transcriptomic profiling results indicated that NB-MSCs were enriched with EMT genes compared to BM-MSCs.

**Conclusions:**

We characterized the biological features, the immunomodulatory capacity and the gene expression profile of NB-MSCs. The NB-MSC gene expression profile and their functional properties suggest a potential role in promoting tumor escape, invasiveness and metastatic traits of NB cancer cells. A better understanding of the complex mechanisms underlying the interactions between NB cells and NB-derived MSCs should shed new light on potential novel therapeutic approaches.

**Electronic supplementary material:**

The online version of this article (10.1186/s12885-018-5082-2) contains supplementary material, which is available to authorized users.

## Background

Neuroblastoma (NB), one of the most common extracranial solid tumors of childhood, arises from embryonic neural crest cells. It may occur anywhere that sympathetic neural tissue is found, but most frequently occurs in the adrenal medulla. Other identifying characteristics of this tumor include the early age of onset, high metastatic disease frequency at diagnosis and the possibility of spontaneous regression [1]. The most aggressive tumors display amplification of the *MYCN* oncogene, which is usually associated with poor survival, even in localized disease.

Like most cancers, the effect of tumor microenvironment on disease progression is not to be underestimate, as highlighted by recent evidence [2–7]. This influence is variable and it is determined by several factors, providing, in the early stages of tumor development, a physical barrier against tumorigenesis, with lymphocytes, macrophages and natural killer (NK) cells playing key roles in tumor suppression [8–10]. During cancer progression, tumor cells may create a supportive milieu that promotes both tumor growth and metastasis by reprogramming the surrounding cells and molecules.

A role for mesenchymal stem cells (MSCs) in the promotion of tumor progression by interacting with tumor cells and other stroma cells in the complex network of the microenvironment has been proposed [11]. It has been demonstrated that NB cells upon pre-incubation with MSCs developed a more invasive behavior towards the bone marrow, primary site of NB metastases. Interestingly, intratumorally injected BM-MSCs reduce NB tumor growth and prolong murine survival, while after systemic administration these cells fail to home and to reach the primary tumor sites [12]; this observation suggests the need for contact between MSCs and NB cells. Moreover, MSCs have been shown to induce invasiveness of NB cells via stimulation of CXCR4 expression both by secretome production and enhanced SDF1/CXCR4 signaling. A crucial role of the CXCL12/CXCR4 axis in promoting the NB invasiveness and the cross-talk of NB cells with the microenvironment has been assessed [13, 14].

The multiple properties of these cells such as self-renewal, differentiation plasticity and ability to modulate immune responses as well as their strong tropism for tumors make them crucial players in the development of a metastatic phenotype.

Little information is available regarding the biological and functional features of MSCs isolated from NB tissue (NB-MSCs) [15]. Therefore, the purpose of this study was to characterize NB-MSCs in terms of morphology, phenotype, differentiation, immunological capacity, proliferative growth and gene expression profile to define their involvement in the tumor microenvironment and in NB progression.

## Methods

### Patients

Seven pediatric patients (3 females and 4 males; median age 27 months, range 18–34 months), diagnosed with NB were enrolled. The NB diagnosis was histologically confirmed. In Table 1, clinical data, Ki67 positive cell percentage and TP53 mutational status are reported. Residual material for histological analysis was used as the starting material for MSC isolation and expansion. Samples were collected prior to chemotherapy. However, in one patient, samples were obtained pre- and post-chemotherapy and in another patient only after chemotherapy treatment.

**Table 1:** Clinical data, neuroblastoma grading and prognostic index of the patients enrolled in the study

Patient	Chemotherapy condition	Tumor localization	MYCN amplification	Tumor stage	INPC^a^	MKI^b^	Ki67 positive cells	TP53
1	Pre	abdominal	yes	IV	unf	> 4	< 10%	mutated
2	Pre	abdominal	yes	I	unf	≤2	> 10%	mutated
3	Pre	abdominal	yes	IV	unf	> 4	> 10%	wild type
4	Pre	adrenal	yes	III	unf	< 2	> 10%	wild type
5	Post	abdominal	yes	IV	unf	> 4	> 10%	mutated
6	Pre	adrenal	no	III	unf	< 2	> 10%	wild type
	Post		yes				< 10%	wild type
7	Pre	abdominal	yes	IV	unf	> 2	> 10%	mutated

^a^*INPC* International Neuroblastoma Pathology Classification, ^b^*MKI* Mitosis-Karyorrhexis Index, *unf* Unfavorable

The study was performed according to the Declaration of Helsinki and with the approval of the Review Board of the Children’s Hospital “G. Di Cristina” (register number 87 Civico 2017). Informed written consent was obtained from the parents and/or legal guardian after receiving information about the study.

### Methods

#### Immunohistochemistry analysis on NB-tissue

In order to characterize the TP53 mutational status and Ki67 proliferation index, which are associated with NB prognosis, we performed an immunohistochemistry analysis on the seven NB patients enrolled in the study. Tissue samples were fixed in 10% neutral buffered formalin for at least 24 h, washed in water for 30 min and placed in ethanol 70% before the automated processing (Leica ASP300 S system). Then, samples were included in High Melt Paraffin (Surgipath Paraplast) and 5 μm thick sections were obtained by using the microtome. For immunohistochemistry analysis of Ki67 and TP53 DO-7 staining, the slides were dewaxed and then subjected to automated retrieval using DAKO PT link (Agilent) in 0.1 M citrate buffer pH 6.0 or pH 9.0 according to the manufacturer’s instructions. Upon overnight exposure at 4 °C to the following primary antibodies, Ki67 (mouse IgG1; Dako Cytomation) and TP53 DO-7 (mouse IgG2b; Novocastra Leica) tissue samples were incubated with biotinylated anti-mouse by using the LSAB 2 Kit (Dako Cytomation or Vectastain kit; Vector) following the manufacturer’s instructions. Staining was detected using 3-amino-9- ethylcarbazole (AEC) chromogen. Nuclei were counter-stained with hematoxylin (Sigma).

### NB-MSC isolation, culture and expansion

MSCs were isolated after tumor tissue mechanical dissociation and collagenase type II treatment as previously described [16]. The cell suspension was then collected and cultured following standard expansion procedure for bone-marrow MSC (BM-MSCs). Briefly, cells were plated in polystyrene culture flasks (Corning Costar, Corning, NY, USA) at a density of 160,000/cm^2^ in D-MEM + GlutaMAX (Gibco) supplemented with 10% FBS (Euroclone), 50 mg/mL gentamicin and 1% penicillin.

NB-MSCs were isolated based on their ability to adhere to plastic as fibroblast-like cells, after being in culture at 37 °C in a humidified atmosphere with 5% CO_2_ and culture medium replacement twice a week. At ≥80% confluence, NB-MSCs were harvested by Trypsin EDTA (Euroclone), replated for expansion at a density of 4000 cells/cm^2^. Previously expanded and characterized BM-MSCs were used as controls for the experiments described in this study.

### Characterization of ex-vivo expanded NB-MSCs

#### Proliferative capacity

Proliferative capacity was defined as cumulative Population Doubling (cPD) calculated with the formula PD = log_10_ (n. of harvested cells/n. of seeded cells)/log_10_2.

#### Flow cytometry and cell cycle analysis

CD73, CD34, CD90, CD14, CD45, CD31, CD105, class I-HLA and HLA-DR, (Beckman Coulter, IL, Milan, Italy) were used for standard MSC characterization, as previously described [17]. Analysis of cell populations was performed with a FACS Navios flow-cytometer (BC).

For stemness marker expression, cells were collected, washed with PBS, fixed and permeabilized where necessary using a Fix and Perm solution (BD Biosciences) for 20 min at room temperature. After washing, samples were stained with primary antibodies against SOX2-AlexaFluor488 (245,610, mouse IgG2a, BD), Nanog-Alexa Fluor488 (N31–355, mouse IgG1k, BD), Oct-3/4-PE (40/OCT3, mouse IgG1k, BD), CD117-PE (104D2, mouse, IgG1k, BD), O4-PE (O4, mouse, IgGM, R&D systems), and corresponding isotype matched controls against CD3 Alexa Fluor 488 (UCTHT1, IgG1k, BD Biosciences), CD4-PE (L200, IgG1k, BD Biosciences) IgM-PE (Goat Anti-Mouse, R&D systems), IgG2A-APC (Mouse, R&D systems). Data were analyzed using AccuriC6 (BD).

Cell cycle analysis was performed on NB-MSCs and BM-MSCs, using 50 μg/ml propidium iodide (Sigma-Aldrich) in 0.1% sodium citrate (Sigma-Aldrich), 0.1% Triton X-100, 10 μg/ml RNAse (Sigma-Aldrich) containing-buffer, after overnight incubation at 4 °C and measured by flow cytometry. Data were analyzed with FlowJo software (Tree Star).

#### Differentiation assays

The ability of NB-MSCs to differentiate into osteoblasts and adipocytes was evaluated, as previously described [18, 19]. For osteogenic differentiation, NB-MSCs were cultured in induction medium, consisting of αMEM, 10% FBS, 10^− 7^ M dexamethasone, 50 mg/ml L-ascorbic acid and 5 mM β-glycerol phosphate (all from Sigma-Aldrich). While adipogenic induction medium consisted of αMEM, 10% FBS, 10-7 M dexamethasone, 50 mg/ml L-ascorbic acid and 5 mM β-glycerol phosphate, 100 mg/ml insulin, 50 mM isobutyl methylxanthine (all from Sigma-Aldrich), 0.5 mM indomethacin (MP Biomedica, Illkirch, France). Medium was changed twice a week and differentiation was evaluated after 21 days. Osteogenic differentiation was evaluated by staining for alkaline phosphatase (AP) activity with Fast Blue and for calcium deposition, with Alizarin Red S staining (both from Sigma-Aldrich). While adipogenic differentiation was demonstrated by the morphological appearance of fat droplets stained with Oil Red O (Bio Optica, Milan, Italy). The ability of NB-MSCs to differentiate in chondrocytes has been evaluated on adherent cells using the hMSC Chondrogenic Differentiation Basal Medium (Lonza), supplemented with TGFβ1 (10 ng/ml), IGF1 (100 ng/ml), transferrin (6.25 μg/ml), insulin (6.25 μg/ml), TGFβ3 (5 ng/ml), 10% FBS and antibiotics (gentamicin and amphotericin) following manufacturing protocol. Briefly, fresh medium was added every 3–4 days. After 21 days, cells were fixed with 2% PFA for 30 min at 37 °C, washed in PBS and stained with Alcian blue for 30 min, than evaluated with phase contrast microscopy.

#### Senescence assay

NB-MSC senescence was assessed by staining with the β-galactosidase (SA-β-gal) staining kit (Cell Signaling Technology, Danvers, MA), according to the manufacturer’s instructions, and evaluated by direct-light microscopy.

#### Immunomodulatory capacity

The ability of NB-MSC to modulate T lymphocytes proliferation was evaluated as previously described [16]. Phytohemagglutinin (4 μg/ml, PHA-P; Sigma-Aldrich) activated PBMCs were cultured in the presence or absence of MSCs at different NB-MSC: PBMC ratios (1:2, 1:20, and 1:200). After 48 h incubation at 37 °C, 5% CO_2_, cultures were pulsed with ^3^H-thymidine (1 μCi/well, specific activity 6.7 μCi/mmole, Perkin Elmer, Waltham, MA) and harvested after 18 h. ^3^H-thymidine incorporation was measured by gamma-counter (PerkinElmer Massachusetts, USA). Experiments were performed in triplicate and results were expressed as Stimulation index (SI = cpm stimulated/cpm unstimulated).

#### NK mediated cytotoxic activity

NK activity was evaluated in effectors recovered from PBMC/MSC mixed cultures in the presence (mixed culture) or absence (ctrl-culture) of MSCs. Briefly, 1 × 10^6^ PBMCs obtained from one healthy donor were cultured in RPMI 1640 (Euroclone, Milan, Italy), 5% autologous plasma and 100 U/ml recombinant IL-2 (Novartis, Varese, Italy) in the presence or absence of NB-MSC or BM-MSC at a 1:10 MSC:PBMC ratio, in 24-well plates for 24 h at 37 °C, 5% CO_2_. Recovered effectors (E) were tested for their capacity to lyse ^51^Cr labelled K562 cells (T) at 100:1, 30:1, 10:1 E:T ratios. After 4 h incubation, 25 μl of the supernatant was collected from each well and counted for 1 min in a gamma-counter (Topcount, Packard, Downers Grove, Illinois). Results were expressed as the percentage of T lysis calculated with the formula: [(experimental release – spontaneous T release)/(total T release – spontaneous T release)] × 100 [20].

#### RNA isolation and real-time PCR

Total RNA was extracted from cell pellets using an RNeasy Mini Kit (Qiagen Inc., Hilden, Germany) and quantitative reverse transcription-PCR (qRT-PCR) was conducted as previously described [21]. In both BM-MSCs and NB-MSCs, the expression of genes related to Stemness, Wnt targets, EMT and Tumor Metastasis was evaluated with the PrimePCR Assay according to the manufacturer’s instructions (PrimePCR Custom Plate, 384-well, Biorad). Collected data were analyzed with the PrimePCR Analysis software (Biorad).

### Statistical analysis

Quantitative data were described as the mean and standard deviation. Statistical significance was determined by analysis of variance (one-way or two ways) with the Bonferroni post-test or the unpaired T-test. Significance was defined as *p* ≤ 0.05. Analyses were performed using the SPSS statistical package (SPSS, Chicago) and Stata 8.0.

## Results

### NB-MSC isolation, expansion and differentiation

**Fig. 1:**
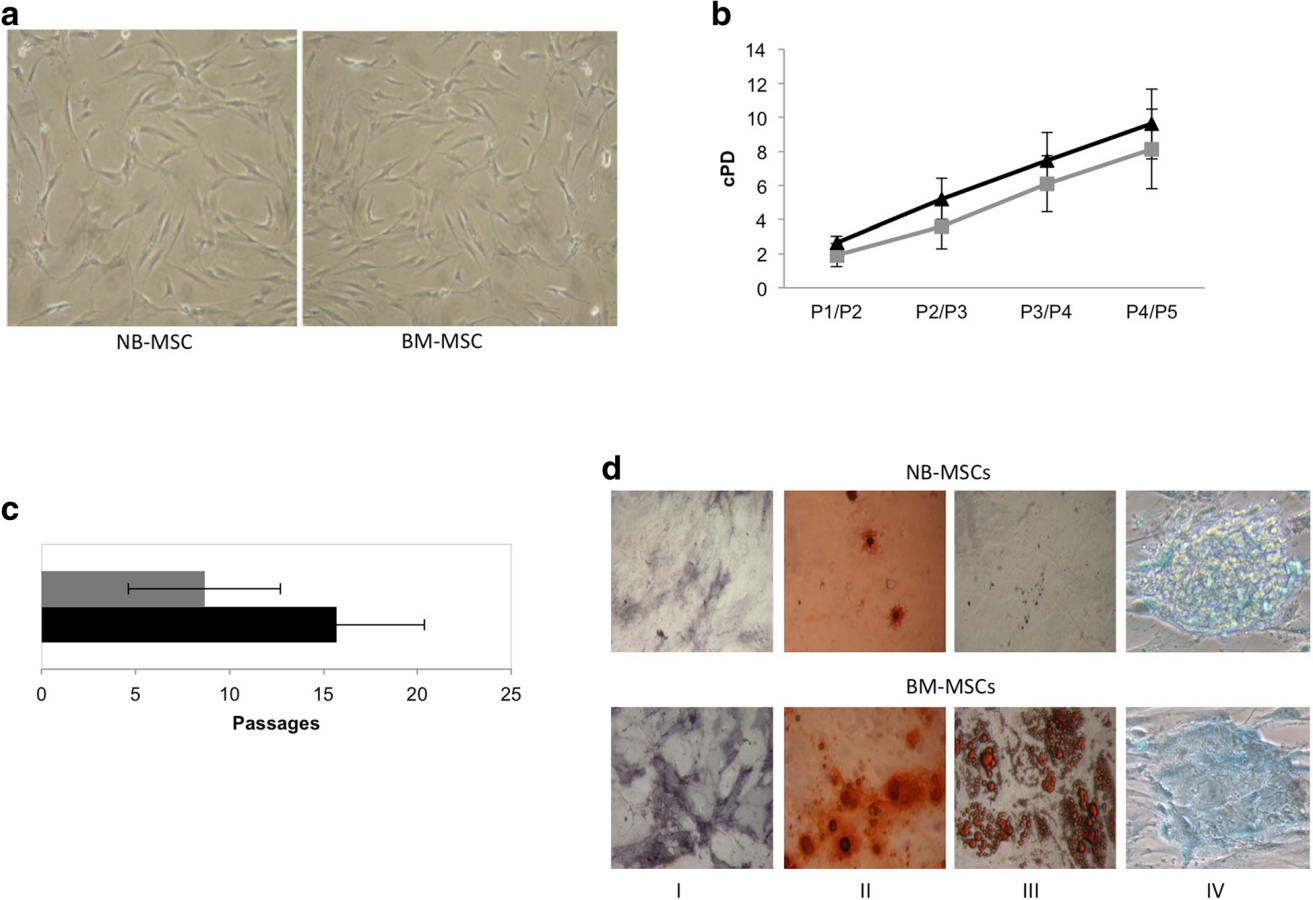
Characterization of ex-vivo expanded NB-MSCs. **a** morphology of culture-expanded MSCs obtained from representative NB-MSC and BM-MSC cultures. MSCs from both the patient and donor display the characteristic spindle-shaped morphology. Original magnification × 4. **b** Cumulative PDs from P1 to P5 of MSCs isolated from BM (black line) and from NB tumor tissue (grey line). The data represent the mean (±SD) of seven NB-MSC and eight BM-MSC cultures. **c** Mean passages (±SD) at which BM-MSC (black bar) and NB-MSC (grey bar) entered senescence. **d** Osteogenic, adipogenic and chondrogenic differentiation capacity of MSCs: the differentiation into osteoblasts is demonstrated by the histological detection of ALP activity (I) and by the histological detection of calcium depositions positive for Alizarin Red S (II); the differentiation into adipocytes is revealed by the formation of lipid droplets stained with oil red O (III); the differentiation into chondrocytes is shown by alcian blue staining (IV). Original magnification 20x

NB-MSCs were isolated and expanded from all NB tissue. NB-MSCs were plastic adherent, displayed the typical spindle shape morphology and a proliferative capacity (Fig. 1a, b). All NB-MSCs reached senescence at an earlier phase compared to healthy donor BM-MSCs (*p* = 0.03) [median passage P7 (range P5-P13) and P15 (range P13-P21), respectively] (Fig. 1c). NB-MSC in vitro differentiation towards different lineages, revealed an osteogenic and chondrogenic differentiation potential similar to BM-MSCs and the lack of an evident adipogenic differentiation capacity (Fig. 1d).

### Phenotypical analysis of NB-MSCs

**Fig. 2:**
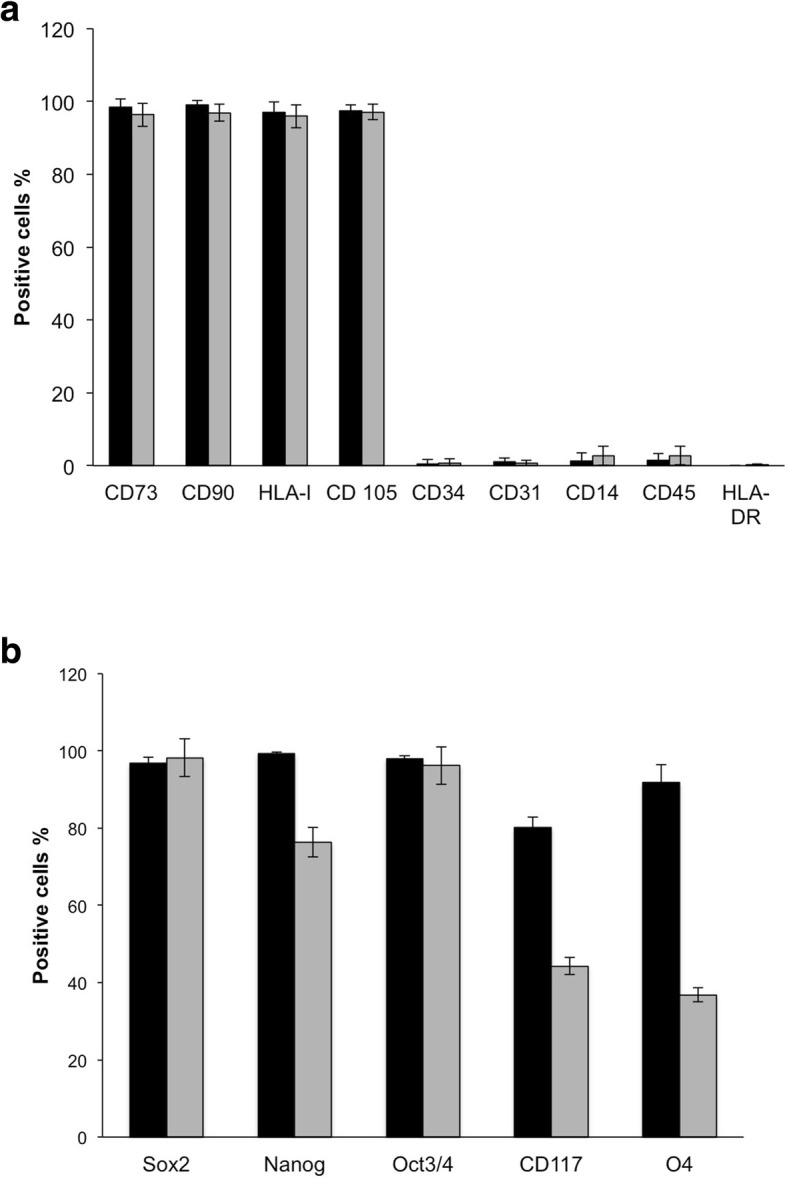
Phenotypical analysis of NB-MSC in comparison with BM-MSC. **a** MSCs are characterized by the expression of the typical surface markers: CD73, CD90, HLA-I, CD105 and the absence of typical hematopoietic cell markers: CD34, CD31, CD14, CD45, and HLA-DR. NB-MSCs and BM-MSCs showed identical phenotypical expression. **b** expression of stemness and differentiation markers Sox2, Nanog, Oct3/4, CD117, O4.Data in (**a**) and (**b**) are expressed as the average percentage of positive cells (BM-MSC: black bar, NB-MSC: grey bar)

NB-MSCs in vitro expanded showed typical expression of cell surface markers; they were positive for CD73, CD90, CD105 and HLA-I and negative for CD34, CD45, CD14, CD31 and HLA-DR (Fig. 2a and Additional file 1: Figure S1). Similar to BM-MSCs, NB-MSCs showed marked expression of stemness markers (Sox2, Nanog, Oct3/4), neuroblastoma stemness marker (CD117), and the oligodendrocyte marker O4 (Fig. 2b and Additional file 2: Figure S2).

### Cell cycle analysis of NB-MSCs

**Fig. 3:**
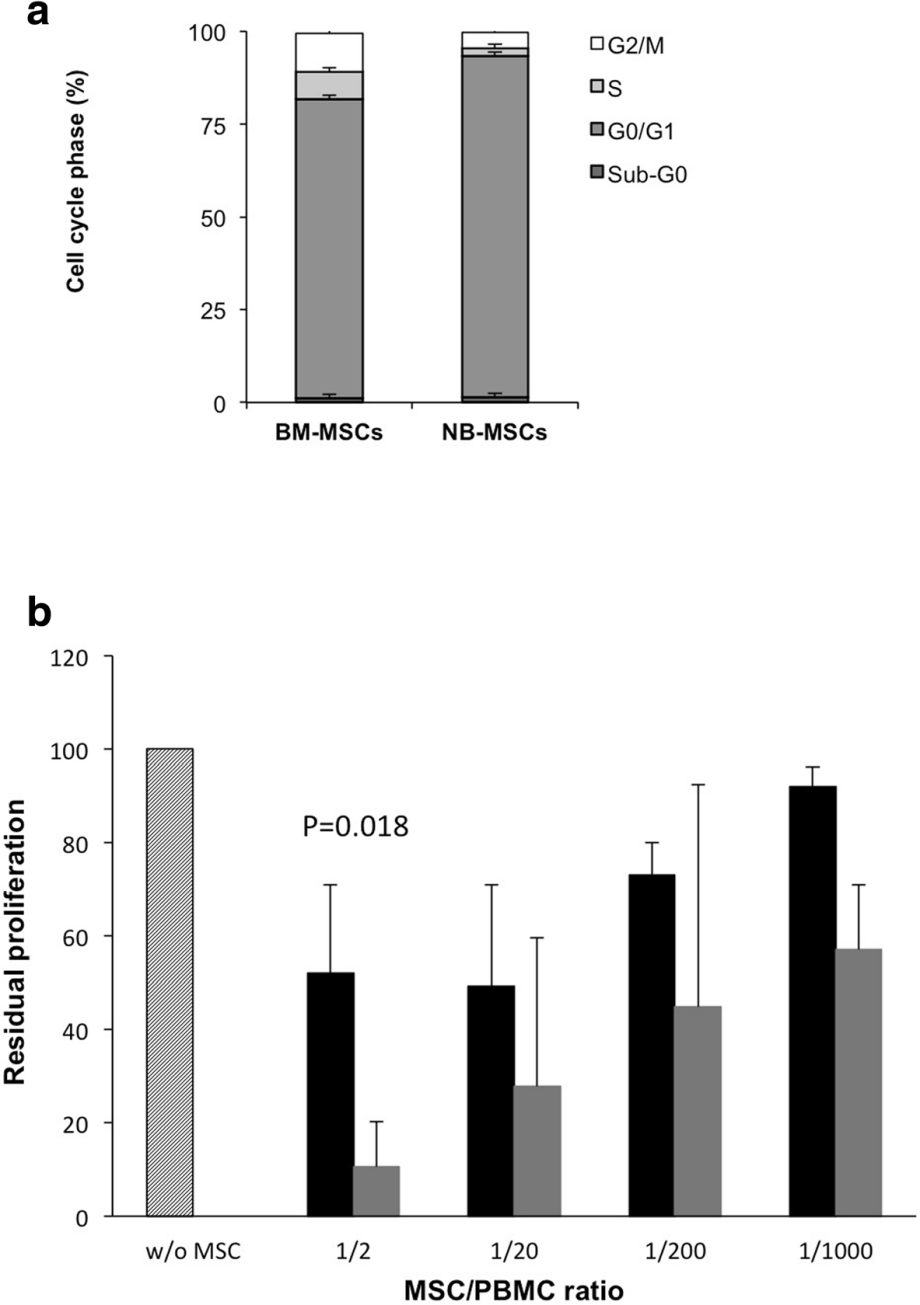
Cell cycle analysis and immunomodulatory effect in 2 BM-MSC and 7 NB-MSC cultures. **a** Cell cycle analysis. The histogram shows the relative cell number percentage in Sub-G0, G0/G1, S, G2/M phases. Data are expressed as the mean ± SD of 3 replicates. **b** In vitro effect of NB-MSCs on PHA-induced PBMC proliferation

NB-MSC cell cycle analysis showed 91% of cells in the G0-G1 phase, 2 and 5% in S and G2/M phase, respectively, while in BM-MSC, 80% of cells were detected in the G0-G1 phase, 7 and 10% in the S-phase and G2/M phase, respectively (p = 0.03) (Fig. 3a and Additional file 3: Figure S3).

### Effect of NB-MSCs on PHA-induced PBMC proliferation and NK activity

Regarding in vitro immunomodulatory capacity on activated T lymphocytes, NB-MSCs resulted more suppressive at a ratio of 1:2 (MSC:PBMC) compared to BM-MSCs (*p* = 0.018) (Fig. 3b). While NK cytotoxic activity, evaluated after co-culture of whole PBMCs with MSCs, was not influenced by co-culture either with BM-MSCs or NB-MSCs.

### Gene expression profiling of NB-MSCs

**Fig. 4:**
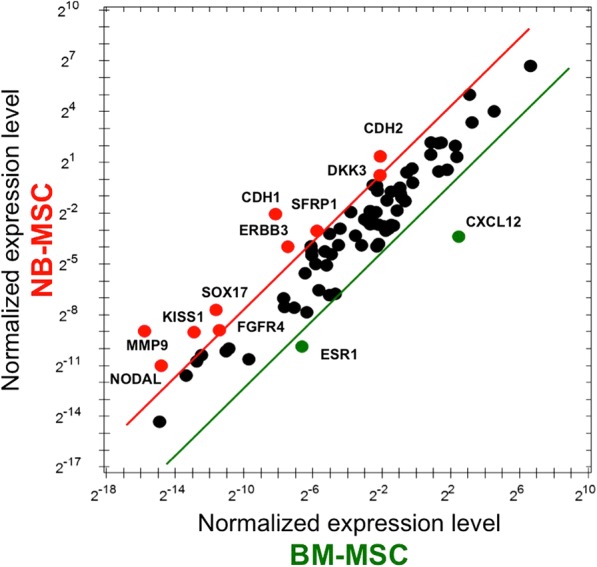
Wnt signaling pathway, stemness and EMT program activation are enhanced in NB-MSCs compared with BM-MSCs. Scatter plot of the genes upregulated in NB-MSCs vs BM-MSCs (5 fold increase) are reported in red, while the genes upregulated in BM-MSCs vs NB-MSCs (5 fold increase) are marked in green. Data are presented as normalized expression values of 3 biological replicates using the PrimePCR Analysis software

Gene expression profiling of Epithelial-to-Mesenchymal Transition (EMT)-, stemness- and Wnt pathway- related genes indicated that approximately 70% of these genes were differently expressed in NB-MSCs compared to BM-MSCs. In detail, 53 genes resulted up-regulated and 27 genes down-regulated as showed in Tables 2 and 3, respectively. In particular, *Cadherin-2 (CDH2)* and *Matrix metallopeptidase 9 (MMP9)* were among the most significantly up-regulated genes in NB-MSC compared with BM-MSC (Fig. 4). These results suggest an enhanced activation of the EMT program and increased migratory capabilities in NB-MSCs that may facilitate their potential role in promoting the invasiveness and metastatic traits of NB cancer cells.

**Table 2:**
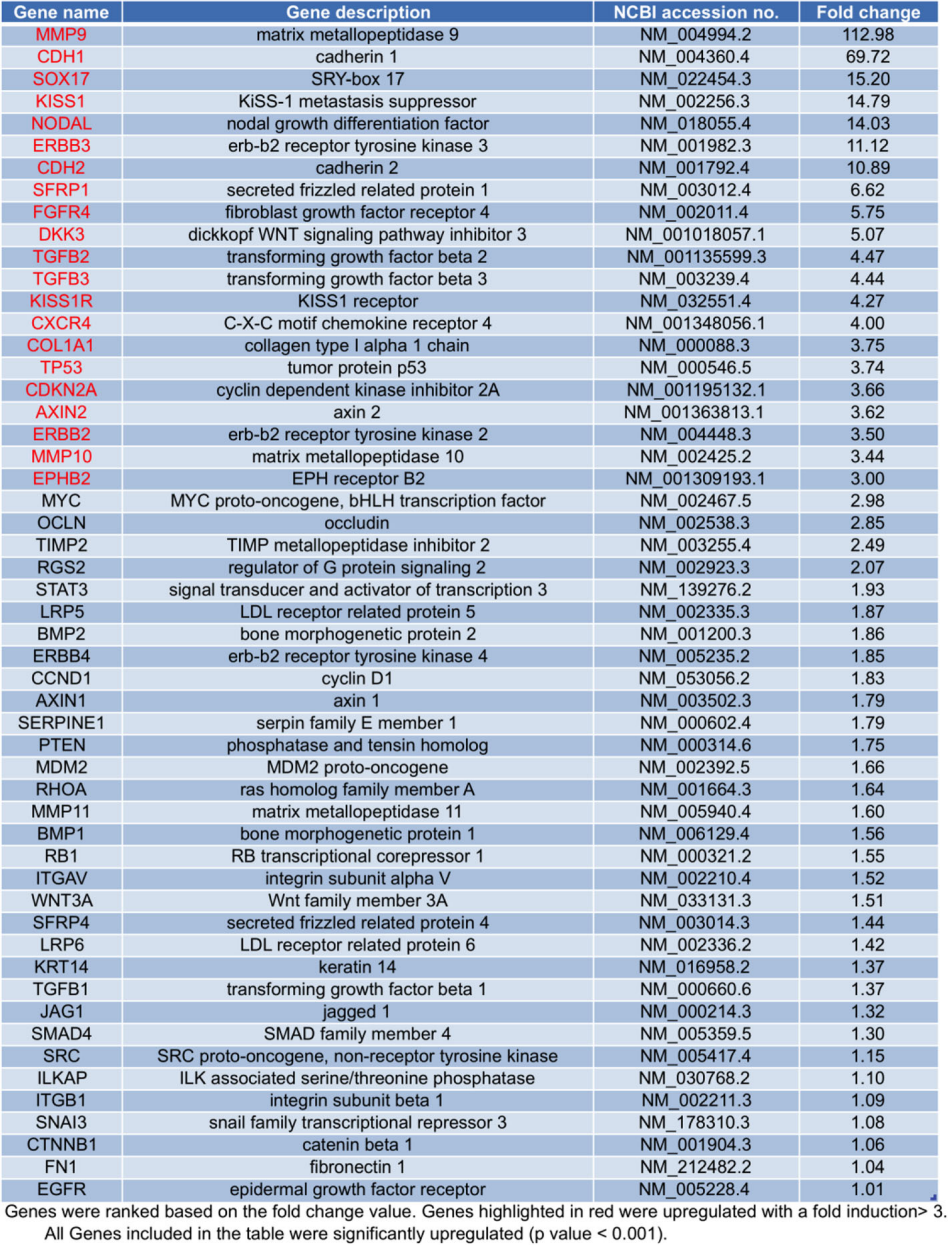
List of upregulated genes in NB-MSCs from NB patients compared with BM-MCs from healthy donors

**Table 3:**
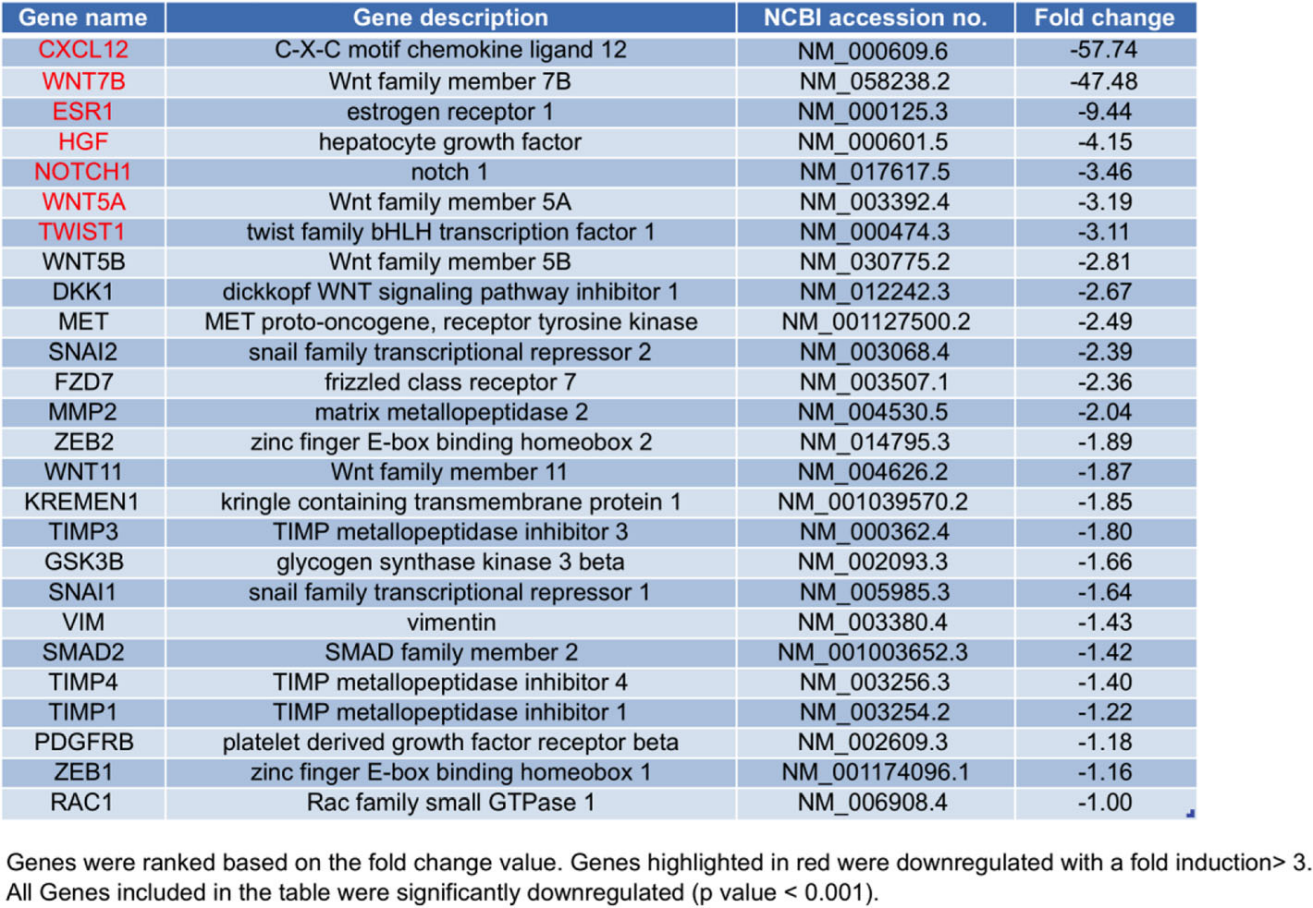
List of downregulated genes in NB-MCs from NB patients compared with BM-MCs from healthy donors

## Discussion

In spite of the different medical interventions available, the NB prognosis remains poor, accounting for over 15% of all pediatric cancer deaths. At diagnosis, about 50% of NB patients have metastases and even a higher percentage suffer from difficult to treat tumors [22–25]. Among these patients only 40% survive long term despite treatment with different therapeutic approaches [26–28]. The interaction of neuroblastoma cancer-initiating cells with their microenvironment has been reported to play an integral role in the maintenance of resistant disease and tumor relapse [2].

The TME primarily consists of MSCs [15]. They are multipotent cells, that can differentiate into several mesenchymal lineages, such as bone, adipose tissue, cartilage, tendon and muscle [29–31]. Additionally, MSCs exert peculiar immunomodulatory effects on several cells involved in the immune response, both in vivo and in vitro, through mechanisms that are not yet completely elucidated [4, 6, 7]. MSCs have been experimentally shown to be influenced by the tumor and subsequently regulate tumor functions. In fact extracellular matrix components have been shown to be influenced by tumor modified MSCs thereby promoting tumor proliferation and metastases [3–7]. As reported by Nakata et al., the exposure of the MSCs to different types of tumor-derived exosomes, including NB, induce the production of pro-tumorigenic cytokines and chemokines, having a direct effect on tumor cell proliferation and survival, on angiogenesis and on the recruitment of other inflammatory cells that favour tumor progression [32].

In the present study, NB-MSCs represent a population that is similar, but not identical, to healthy donor BM-MSCs. In detail, they exhibit different adipogenic differentiation and immunomodulatory capabilities. Moreover, NB-MSC gene expression profiling results suggest their potential role in promoting the invasiveness and metastatic traits of NB cancer cells.

Under standard conditions, we isolated and propagated MSCs derived from pediatric NB tissue at disease onset, and in two cases after administration of chemotherapy. MSCs isolated and propagated from pediatric NB tissue displayed the typical MSC morphology and phenotype [33] and proliferative capacity superimposable to BM-MSCs. The similar expression levels of stemness markers, such as Nanog, OCT3/4 and SOX2, observed both in NB-MSCs and BM-MSCs, suggest that NB-MSCs, even when derived from the tissue/tumor microenvironment, maintain BM-MSC multipotentiality [30, 31].

In the cell cycle analysis, NB-MSCs showed an increased number of cells in the G0-G1 phase compared to BM-MSCs. It has been reported that a high content of G1 phase-blocked cells in cancer might imply a still unclear situation defined as “dormancy” that poses fundamental questions, which extend beyond the cancer proliferation/suppressor balance in primary cancer [34]. In fact, dormant tumor cells are characterized by G0/G1 phase arrest and chemotherapeutic drugs resistance [34]. Our observation of NB-MSCs arrest in G0/G1 cell cycle phase could support the essential role of MSCs, in regulating cancer dormancy [35, 36]. These data provide a valuable tool to understand the MSC role in tumorigenesis and therefore open new therapeutic avenues for the prevention of cancer recurrence.

Regarding in vitro differentiation capabilities, NB-MSCs revealed a similar osteogenic and chondrogenic differentiation potential compared with BM-MSCs; on the contrary, NB-MSCs did not differentiate into adipocytes. Even though, tumor-MSCs exhibit varying differentiation capabilities [37], according to our results, only a small subset of MSCs isolated from pediatric neuroblastoma, teratoma, Ewing sarcoma, and rhabdomyosarcoma specimens may be induced to differentiate into adipocytes while they respond to osteogenic induction similarly to BM-MSCs [34]. Nevertheless, the lack of evident adipocytic differentiation together with an enhanced EMT-related gene expression profile and a different immunological response, lead to the hypothesis that NB-MSCs may be already dysregulated. However, further studies are needed to determine whether these cells could be defined as “cancer stem cells”.

The immunomodulatory properties of MSCs have been demonstrated using different in vitro and in vivo study approaches. MSCs regulate immunity by interacting with innate immune cells (including macrophages, NK cells, and dendritic cells), and adaptive immune cells (including B and T cells) [38–40]. In the present study, we report more evident NB-MSC anti-proliferative effects on immune cells compared with BM-MSCs, confirming the impact of the tumor microenvironment on MSC functions [7]. It is well known that NK cells are potent anti-tumor cells [41] and possess strong cytotoxic activity against NB, both in vitro [42] and in vivo [43]. Johann et al. [15] have reported that NK cytotoxicity is significantly impaired after co-culture with tumor stromal cells. Galland et al. [10] have demonstrated that squamous cell lung carcinoma derived-MSCs exert a more pronounced immunosuppressive effect on NK cell functions and phenotype compared with the non tumor derived-MSCs through different mechanisms. In our study, the NK cytotoxic capability was not influenced by co-culture either with BM-MSCs or with NB-MSCs. We believe that these contrasting data may be ascribed to the different culture conditions and the different starting population, purified NK or whole PBMCs.

Abnormal regulation of TP53 has a critical role in tumorigenesis. Velletri et al. [44] reported that MSC immunomodulatory properties may be influenced by the TP53 mutational status, which leads to genome instability and subsequent functional alterations. About 50% of our NB patients resulted TP53 mutated, in disagreement with data reported in the literature, where 2% of patients are mutated. Even if these results may depend on the small number of patients, we believe that this mutational status could affect the properties of NB-MSCs and tumor microenvironment and dictate the conditions for cancer development and progression [44]. Nevertheless, it is important to consider that although TP53 is rarely mutated in NB, it is functionally inactivated in the majority of both MYCN amplified and MYCN WT subsets NB [45–47].

It has been reported that tumor-derived MSCs may promote cell invasiveness, migration and EMT of cancer cells in many tumors [48, 49]. Recently, Rodriguez-Milla et al. identified about 500 genes differentially expressed in BM-MSCs from NB patients and BM-MSCs from normal donors. Of note, genes involved in many biological processes including neurological system process, apoptosis, cell adhesion, cell surface receptor and intracellular signaling were up-regulated while genes related to the immune response were down-regulated. These findings suggest a dysregulation of crucial cellular pathways in MSCs in response to NB tumor [50].

Here, we found that the transcriptomic profiling of NB-MSCs isolated from tumor samples derived from NB patients was enriched in EMT-associated genes compared to BM-MSCs, in particular in CDH2 and MMP-9 genes. It is known that CDH2, also termed N-cadherin, favors transendothelial migration, and MMP-9, a key enzyme in extracellular matrix remodeling, promotes cell invasion and metastasis by degrading collagens and fibronectin. Moreover, high expression levels of CDH2 are associated with a poor prognosis in NB and MMP-9 is highly expressed in high-risk NB tumors [51–53]. Furthermore, in our gene expression profiling data NB-MSCs express higher levels of CXCR4 while down-regulate CXCL12 expression levels compared with BM-MSCs, supporting the pivotal role of CXCL12/CXCR4 axis in promoting NB invasiveness.

All together, these data provide evidence supporting a role for NB-MSCs in facilitating the metastatic process of NB cells, although further in vivo studies are needed to demonstrate this hypothesis. We recognize that this study has some limitations. The sample size was small, but consistent with that of other studies investigating this specific population. Additionally, the follow-up of patients was too limited to be significant and provide correlations. Despite these limitations, our findings support a role for NB-MSCs as a crucial factor on the microenvironmental regulation of tumor progression and metastasis. Further studies analyzing a broader number of samples are mandatory to confirm these results.

## Conclusions

We describe a phenotypic and functional analysis of NB-MSCs expanded from tumor tissue in comparison with healthy BM-MSCs. NB- and BM-MSCs share important similarities, however, there are some significant differences in terms of functional properties and gene profile, that suggest their potential role in promoting tumor escape, invasiveness and metastatic traits of NB cancer cells.

A better understanding of the complex mechanisms underlying the interactions between NB cells and NB-derived MSCs should shed light on potential novel therapeutic approaches such as the use of MSCs as a tool for drug-delivery.

## Additional files

Additional file 1: **Figure S1.** Immunophenotype characterization of NB-MSCs. Immunophenotype characterization of NB tissue derived-MSC from a representative sample. NB-MSCs are gated on physical parameter (FSC and SSC). Surface marker expression of NB-MSC are reported in overlay histograms with light grey peaks representing negative control by isotype-matched, nonreactive fluorochrome-conjugated antibodies. Dark grey peaks represent positive cells. Histograms of surface marker expression are typical of MSC being positive for CD105, CD73, CD90 and HLA-I and negative for HLA-DR, CD31, CD14, CD45 and CD34. (JPG 132 kb)

Additional file 2: **Figure S2.** Flow cytometry profiles of selected stemness markers in BM-MSCs and NB-MSCs. (Left panel) representative scatter plots of SSC vs FSC of BM-MSC and NB-MSC cells. (Right panel) indicative flow cytometry profiles of selected markers in BM-MSC and NB-MSC samples. Dotted grey light histograms represent the relative isotype matched control. (JPG 157 kb)

Additional file 3: **Figure S3.** Flow cytometry analysis in BM-MSCs and NB-MSCs. Flow cytometry analysis of cell cycle in BM-MSCs and NB-MSCs. Plots show the percentage of cells in sub-G0 phase (white box), G0-G1 phase (grey box), S phase (pink box) and G2-M phase (light yellow box). (JPG 132 kb)

## Abbreviations

BM-MSC: Mesenchymal stromal cells isolated from healthy donor bone marrow
CDH 2: Cadherin-2
cPD: Cumulative Population Doubling
EMT: Epithelial-mesenchymal transition
MMP9: Matrix metallopeptidase 9
MSC: Mesenchymal stromal cells
NB: Neuroblastoma
NB-MSC: Mesenchymal stromal cells isolated from neuroblastoma tissue
NK: Natural killer
PBMCs: Peripheral blood mononuclear cells
PHA: Phytohemagglutinin
pRT-PCR: Quantitative reverse transcription-PCR
