# Supplementary figures and images for: Microenvironment in neuroblastoma: isolation and characterization of tumor-derived mesenchymal stromal cells
Source: BMC Cancer. 2018 Nov 27;18:1176. doi: 10.1186/s12885-018-5082-2 (PMC6260687; doi:10.1186/s12885-018-5082-2)

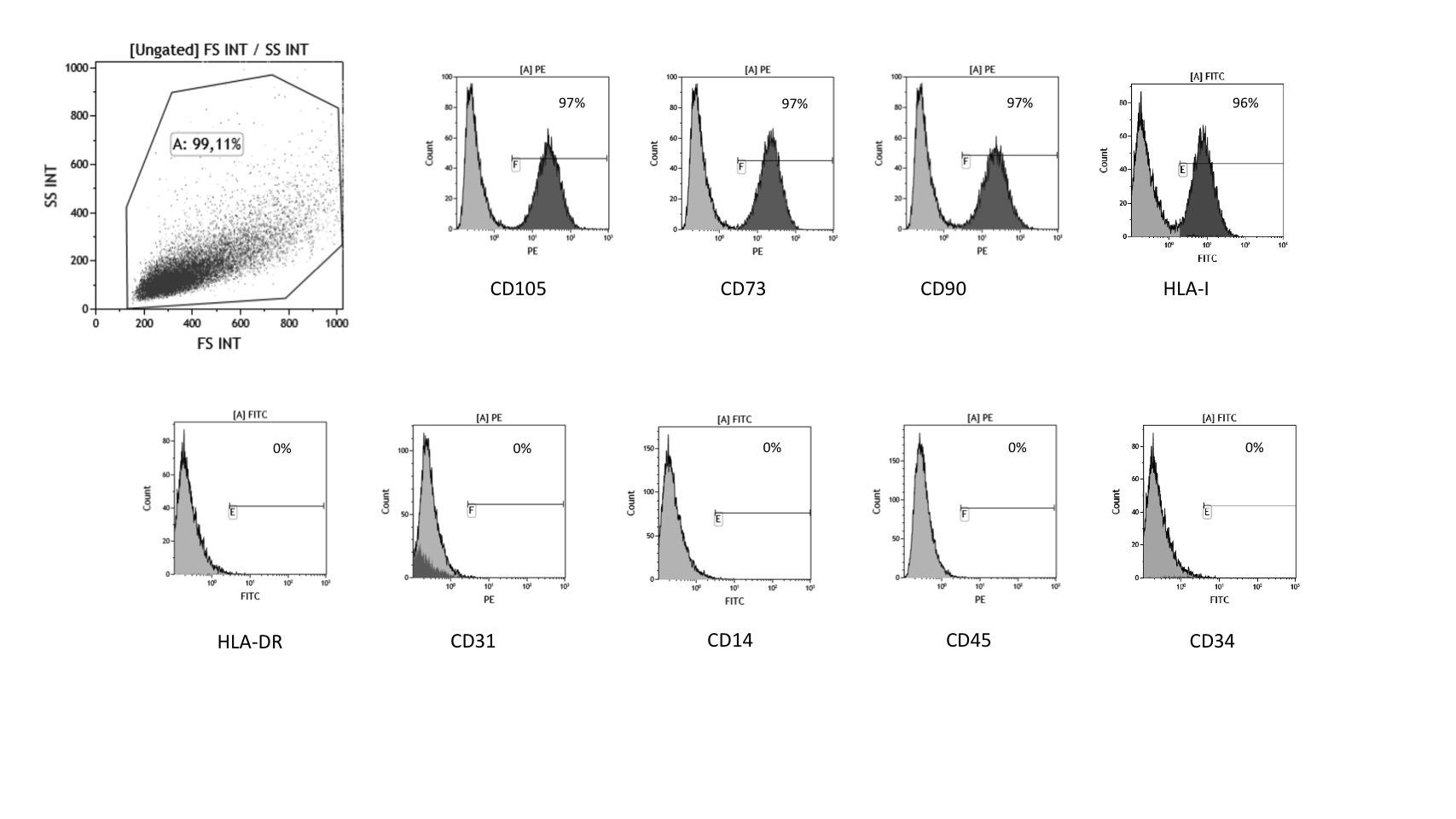

Supplement: Supplementary file 1 — Figure S1. Immunophenotype characterization of NB-MSCs. Immunophenotype characterization of NB tissue derived-MSC from a representative sample. NB-MSCs are gated on physical parameter (FSC and SSC). Surface marker expression of NB-MSC are reported in overlay histograms with light grey peaks representing negative control by isotype-matched, nonreactive fluorochrome-conjugated antibodies. Dark grey peaks represent positive cells. Histograms of surface marker expression are typical of MSC being positive for CD105, CD73, CD90 and HLA-I and negative for HLA-DR, CD31, CD14, CD45 and CD34. (JPG 132 kb) [file 12885_2018_5082_MOESM1_ESM.jpg]

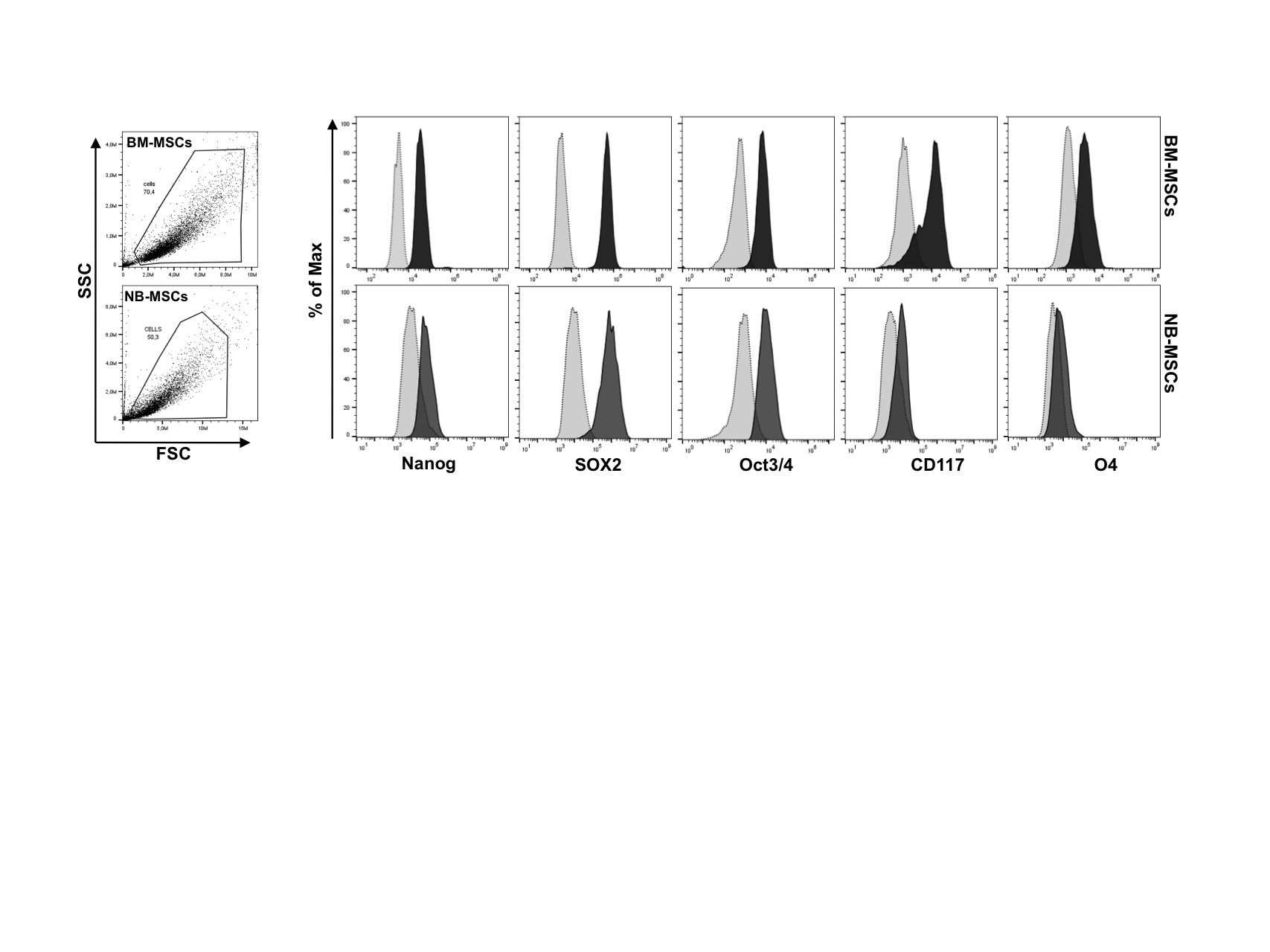

Supplement: Supplementary file 2 — Figure S2. Flow cytometry profiles of selected stemness markers in BM-MSCs and NB-MSCs. (Left panel) representative scatter plots of SSC vs FSC of BM-MSC and NB-MSC cells. (Right panel) indicative flow cytometry profiles of selected markers in BM-MSC and NB-MSC samples. Dotted grey light histograms represent the relative isotype matched control. (JPG 157 kb) [file 12885_2018_5082_MOESM2_ESM.jpg]

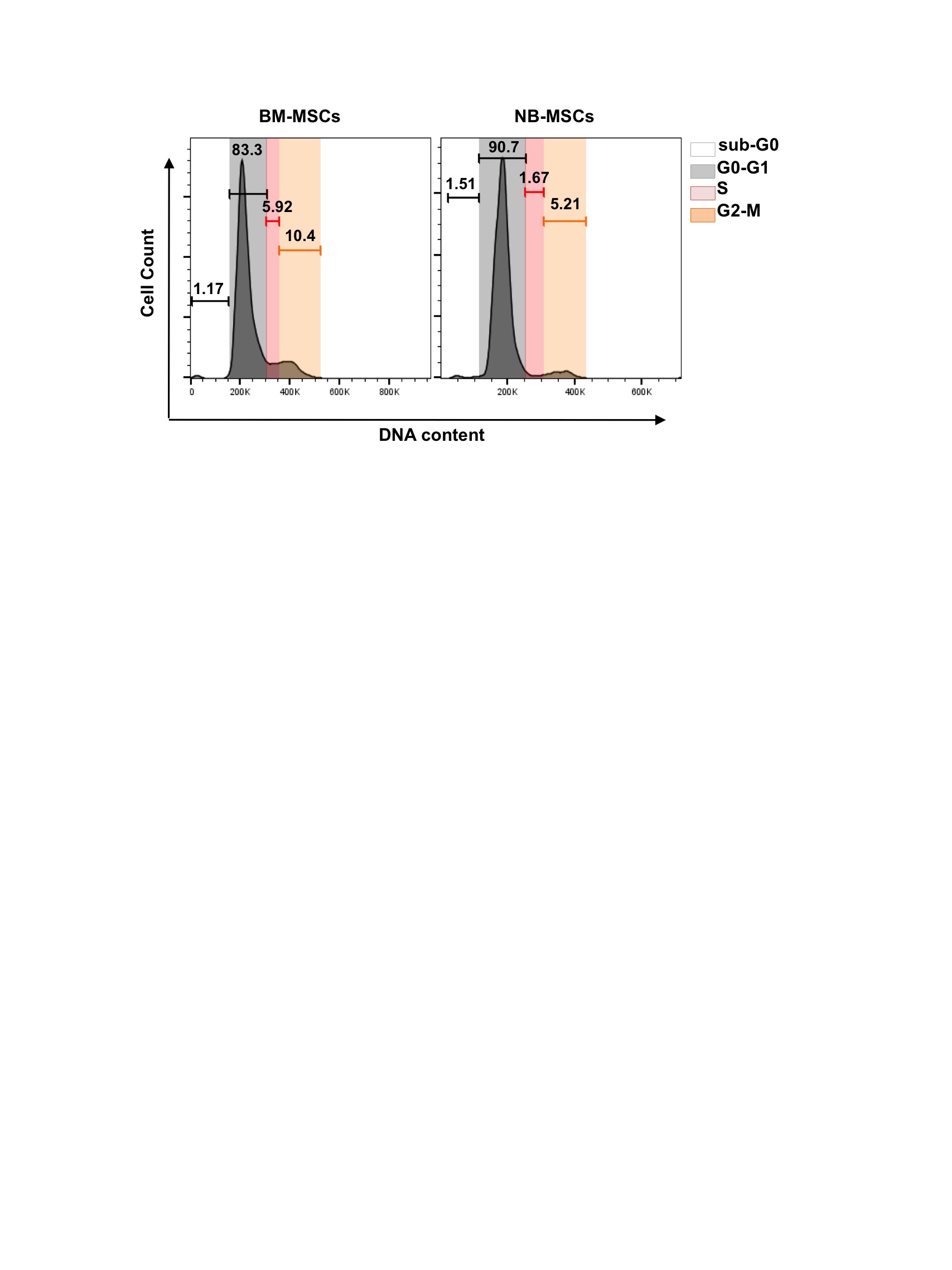

Supplement: Supplementary file 3 — Figure S3. Flow cytometry analysis in BM-MSCs and NB-MSCs. Flow cytometry analysis of cell cycle in BM-MSCs and NB-MSCs. Plots show the percentage of cells in sub-G0 phase (white box), G0-G1 phase (grey box), S phase (pink box) and G2-M phase (light yellow box). (JPG 132 kb) [file 12885_2018_5082_MOESM3_ESM.jpg]
